# ALDH1A3 in CSCs

**DOI:** 10.18632/aging.101236

**Published:** 2017-05-03

**Authors:** Kelly E. Sullivan, Richard A. Cerione, Kristin F. Wilson

**Affiliations:** Department of Molecular Medicine, Cornell University, Ithaca, NY, USA

**Keywords:** ALDH1A3, tissue transglutaminase, cancer stem cell, glioma stem cell, retinoic acid

In recent years, a sub-population of cancer cells referred to as cancer stem cells (CSCs) has come under intense investigation for its potential roles in tumor initiation, therapy resistance, and tumor recurrence in a variety of neoplasias. These tumor-initiating cells share a number of characteristics with their normal stem cell counterparts, including the capacity to self-renew, differentiate into diverse cell types, and express molecular markers such as aldehyde dehydrogenase (ALDH), SOX2, CD133, CD44, and ABCG2. In particular, the activity of ALDH1 subfamily isozymes has been routinely used to isolate CSCs from whole tumors using flow cytometry, both alone and in combination with other markers. However, despite the use of ALDH1 activity as a CSC marker for several years, and evidence for its role in maintaining the stemness of mesenchymal glioma stem cells (MES GSCs) [1], very little is known about the specific mechanisms by which ALDH1 enzymes contribute to the stem cell/cancer stem cell phenotype. We have recently proposed that the retinaldehyde dehydrogenase activity of ALDH1A3, which converts retinal to retinoic acid (RA), a transcriptional activator, may contribute to the aggressive phenotype of MES GSCs [2]. More than 500 genes have been previously suggested to be regulated, either directly or indirectly, by RA [3]; thus, we hypothesized that RA-regulated genes induced downstream of ALDH1A3 activity may play a role in establishing a unique transcriptional profile that favors the stem cell/cancer stem cell phenotype. Uncovering this gene expression pattern may provide not only a deeper understanding of how CSCs fuel neoplastic growth and therapy resistance, but may also present novel therapeutic targets for eliminating this highly resistant population of cells.

While investigating potential transcriptional targets downstream of ALDH1A3 and RA in GSCs, we determined that tissue transglutaminase (tTG), a dual-function GTPase/acyltransferase encoded by the RA-responsive gene *TGM2*, is selectively expressed in ALDH1A3^+^ GSCs, but not in ALDH1A3^−^ GSCs [2]. Moreover, the knockdown of ALDH1A3 is accompanied by a subsequent decline in tTG mRNA, which can be rescued by the addition of RA. These results suggest that *TGM2* is indeed sensitive to ALDH1A3 activity, likely directly through the retinoic acid response elements in its promoter region. As further evidence for this regulatory pathway, we have demonstrated in ALDH1A3^−^/tTG^−^ GSCs that the ectopic expression of wild type ALDH1A3, but not a catalytically inactive form, leads to the expression of tTG. This relationship between ALDH1 activity and tTG expression was correlated in other cancer cell lines as well, including cells derived from cervical, lung, and pancreatic carcinomas, and may pertain to a wide variety of cancers [2].

The relationship between ALDH1A3 and tTG is significant because tTG is linked to the development of aggressive cancers, and has been shown to play a role in chemotherapy resistance, migration, and survival of cancer cells, and additionally impacts the ability of CSCs to self renew [4]. Thus, ALDH1A3 contributes to the insidious CSC phenotype, at least in part, through its ability to regulate the expression of this important survival factor in cancer. It also highlights the opportunity to target tTG as a strategy to eliminate ALDH1A3^+^ CSC populations, including GSCs. Conventional inhibitors of tTG target the transamidation site of the enzyme, thereby inhibiting the ability of tTG to function as an acyltransferase. It is now appreciated, however, that such inhibitors can be extremely effective in promoting cell death regardless of whether tTG is a functional acyltransferase in any given cellular context, by their ability to induce a global conformational change in tTG [5, 6]. Specifically, upon drug binding, tTG transitions from a closed, globular structure to an open, elongated structure that is highly toxic to cells. Although this conformation is naturally-occurring and required for the transglutaminase activity when tTG is secreted from cells, the conformation is strictly regulated within cells. It is the binding of GTP/GDP which induces tTG to adopt a closed conformation, whereas the release of guanine nucleotide and high concentrations of Ca^2+^ allow tTG to adopt the open, acyltransferase-active state. Based on the high cellular levels of GTP and the carefully regulated secretion of Ca^2+^, it is likely that tTG primarily exists in the closed state in cells. Thus, we and others have been interested in developing new classes of compounds that function to stabilize the open conformation of tTG, as there is an important need for next generation tTG inhibitors with enhanced specificity and cell permeability to allow the targeting of tTG to become a clinically relevant therapeutic strategy.

Our study sheds light on just one of potentially hundreds of transcriptional targets of RA downstream of ALDH1 activity, and highlight the need to further define the gene expression signature that accompanies this CSC marker. RNA-Seq experiments in ALDH1^+^ CSCs derived from a diverse array of cancers may help to pinpoint common transcriptional targets of RA in these cells, and may also identify additional therapeutic targets that can resensitize recurrent or refractory cancers to standard therapies.
